# Increasingly strong reduction in breast cancer mortality due to screening

**DOI:** 10.1038/bjc.2011.44

**Published:** 2011-02-22

**Authors:** G van Schoor, S M Moss, J D M Otten, R Donders, E Paap, G J den Heeten, R Holland, M J M Broeders, A L M Verbeek

**Affiliations:** 1 Department of Epidemiology, Biostatistics and HTA, Radboud University Nijmegen Medical Centre, PO Box 9101, 6500 HB Nijmegen, The Netherlands; 2 Cancer Screening Evaluation Unit, Institute of Cancer Research, 15 Cotswold Road, Sutton, Surrey 2M2 5NG, UK; 3 National Expert and Training Centre for Breast Cancer Screening, PO Box 6873, 6503 GJ Nijmegen, The Netherlands

**Keywords:** breast cancer, breast cancer mortality, screening, case–referent study

## Abstract

**Background:**

Favourable outcomes of breast cancer screening trials in the 1970s and 1980s resulted in the launch of population-based service screening programmes in many Western countries. We investigated whether improvements in mammography and treatment modalities have had an influence on the effectiveness of breast cancer screening from 1975 to 2008.

**Methods:**

In Nijmegen, the Netherlands, 55 529 women received an invitation for screening between 1975 and 2008. We designed a case–referent study to evaluate the impact of mammographic screening on breast cancer mortality over time from 1975 to 2008. A total number of 282 breast cancer deaths were identified, and 1410 referents aged 50–69 were sampled from the population invited for screening. We estimated the effectiveness by calculating the odds ratio (OR) indicating the breast cancer death rate for screened *vs* unscreened women.

**Results:**

The breast cancer death rate in the screened group over the complete period was 35% lower than in the unscreened group (OR=0.65; 95% CI=0.49–0.87). Analysis by calendar year showed an increasing effectiveness from a 28% reduction in breast cancer mortality in the period 1975–1991 (OR=0.72; 95% CI=0.47–1.09) to 65% in the period 1992–2008 (OR=0.35; 95% CI=0.19–0.64).

**Conclusion:**

Our results show an increasingly strong reduction in breast cancer mortality over time because of mammographic screening.

Breast cancer screening trials conducted in the 1970s and 1980s demonstrated a 20–30% reduction in breast cancer mortality for women aged 50–69 (Nelson *et al*, 2009). On the basis of these trial results, service screening programmes for breast cancer were implemented on a large scale in Europe, North America and Australia in the 1990s (Shapiro *et al*, 1998). Subsequent evaluations of these programmes have shown a beneficial effect on breast cancer mortality, which has been comparable with the trial outcomes (Demissie *et al*, 1998; Gabe and Duffy, 2005).

Since the trial era and the start of service screening, major advances have been made in the detection and treatment of breast cancer (Early Breast Cancer Trialists' Collaborative Group, 2005; Yaffe *et al*, 2008). The complete screening chain, from the technical aspects of mammography to the training and experience of radiographers and radiologists has improved. In addition, since the 1980s there has been a growing use of adjuvant therapy. No study has yet evaluated the influence of screening on breast cancer mortality taking into account the developments in screening performance and treatment over time.

Trends show a decline in breast cancer mortality. Some investigators have attributed this to screening and improved treatment (Levi *et al*, 2005; Héry *et al*, 2008; Otten *et al*, 2008; Autier *et al*, 2010), while others suggested that screening was not relevant (Zahl and Maehlen, 2005; Becker *et al*, 2007; Jorgensen *et al*, 2010). Although useful and important, the analysis of trends in breast cancer mortality should be interpreted with caution for inference on causal relations. Breast cancer mortality is also declining in age groups not invited for screening and in countries without a national screening programme (Autier *et al*, 2010).

To achieve a reliable assessment of the effect of screening on mortality, it is necessary to make a direct link between a woman's cause of death and her screening history (Verbeek and Broeders, 2010). Data from long-running screening programmes are limited, except in Nijmegen, the Netherlands, where a programme for breast cancer service screening was started in 1975 (Holland *et al*, 2007). Between then and 2008, 405 131 invitations were sent to 55 529 women aged 35 years and older.

We have used data from this ongoing programme to investigate the impact of screening on breast cancer mortality between 1975 and 2008.

## Materials and Methods

### Setting

We designed our study based on the population of women invited to the service screening programme in Nijmegen, the Netherlands. In 1975, this programme started inviting women aged 35 years and over for a biennial mammographic screening examination. In 1989, at the start of the national screening programme, the age of invitation was gradually adapted to that of the national policy, which at that time was 50–69 years until 1997, and 50–74 years from 1998 onwards. More than 257 300 screening examinations were performed up to 2008. The screening examination consisted of a two-view mammogram (a mediolateral oblique and craniocaudal view) in initial screens. In subsequent screenings, the mediolateral oblique view is standard. Additional craniocaudal views are performed only on indication, for example, dense glandular tissue, implants and whenever abnormalities are suspected by the radiographer. At present, craniocaudal view is conducted in about 50% of the women during subsequent examination. A detailed description of the programme has been published (Otten *et al*, 1996).

A separate registry holds information on all patients with breast cancer in Nijmegen diagnosed within and outside the screening programme. Vital status was obtained from the Municipal Personal Records Data Base (GBA) up to and including 2008. Assessments of causes of death were made by a committee of physicians comprising a pathologist, medical oncologist and a radiologist. The committee members were unaware of the screening history. Both our screening and patient datasets are registered with the Netherlands Data Protection Authority.

### Study design and study population

We applied a case–referent design (Verbeek and Broeders, 2010) to evaluate the effect of mammographic screening on breast cancer mortality by calendar year of invitation. Previous evaluations of screening have used the case–control design (Gabe and Duffy, 2005). We prefer the term case–referent study to case–control study in this context because the uptake of screening in the case group of breast cancer deaths is referred to the probability of having been screened in the population from which the cases originate. The lack of overlap in the age groups over calendar time prompted us to restrict the study population to women aged 50–69 at invitation.

In the case series of breast cancer deaths, we ascertained whether women were screened or not screened before breast cancer diagnosis, and calculated the odds of having been screened in this period. To interpret the screening odds in the case group, we also calculated the screening odds in a reference group. For each case, five referents were randomly sampled from the population of women invited for screening. Referents had to be eligible for screening, they did not have breast cancer at the time of invitation and were living in Nijmegen at the time of death of the case. This type of sampling follows the principle of incidence density sampling (Miettinen, 1976; Greenland and Thomas, 1982). The purpose of the case–referent design is to arrive at a valid estimate of the breast cancer mortality rate in both the screened and unscreened population.

### Relevant time frame for screening

Screening can only be effective if the examination is performed in the period that breast cancer is developing and potentially detectable by the screening test before symptoms appear (Weiss *et al*, 1992; Broeders and Verbeek, 2005). The duration of the detectable preclinical period is unknown at the individual level; based on estimates of lead time for breast cancer (Weiss *et al*, 1992; Broeders and Verbeek, 2005) we have set the time frame for screening invitation at a 4-year period before breast cancer diagnosis of the case. In a biennial screening schedule, this period includes two consecutive invitations, that is, the index-invitation (the most recent invitation before diagnosis of the case) and the screening preceding the index. The year of index-invitation is the calendar year of the date of the invitation to the index-screening. The age at index-invitation is the age at this point in time. Both cases and referents have had the same opportunity for screening; therefore exposure to screening is defined as having been screened or not in the 4-year period.

As a result of this 4-year time frame and the constant participation in our programme, there are equal numbers of initial and subsequent screening examinations in our study population over time.

### Analysis

To estimate the effect of screening on breast cancer mortality, we calculated the odds ratio (OR), using logistic regression techniques (Rothman *et al*, 2008). The OR is the odds of having been screened *vs* not screened in the case series of breast cancer deaths, compared with the odds in the reference group from which the cases theoretically originate. As such, the OR is the breast cancer mortality in screened women divided by the breast cancer mortality in unscreened women (Miettinen, 1976).

First, the OR was calculated for the entire screening era from 1975 through 2008. Second, we calculated the ORs in the calendar periods 1975–1991 and 1992–2008. In order to make sure that the two groups were followed for an equal amount of time, we restricted this part of the analysis to cases who died within the same calendar period. Finally, the effect by calendar year (continuous variable) at index-invitation was assessed by including an interaction term, the combination of screening and calendar year, in the logistic regression model. We corrected the ORs for the confounding influence of age at index-invitation by stratification into 5-year age groups. SAS 9.2 software (SAS Institute Inc., Cary, NC, USA) was used for the analysis.

## Results

### Characteristics of the study population

Between 1975 and 2008, a total number of 282 breast cancer deaths were identified. We randomly sampled 1410 referents from the population invited for screening in the same period. The median age at index-invitation in the case group was 59 (interquartile range 54–64) and 57 (interquartile range 53–62) in the reference group.

### Screening effect

Over the entire screening period from 1975 to 2008, 191 cases were screened and 91 not screened, 1089 referents were screened and 321 not screened. After correction for the confounding influence of age at invitation, the screened women experienced a 35% lower breast cancer mortality rate compared with unscreened women (OR=0.65; 95% CI=0.49–0.87; Table 1).

### Impact of calendar period

Among women invited between 1975 and 1991, screening prevented 28% of the otherwise prevailing breast cancer mortality (OR=0.72; 95% CI=0.47–1.09; Table 1). In the period 1992–2008, the breast cancer mortality was 65% lower in screened women compared with unscreened women (OR=0.35; 95% CI=0.19–0.64); *P*-value for the interaction between period and screening effect=0.04.

**Figure 1:**
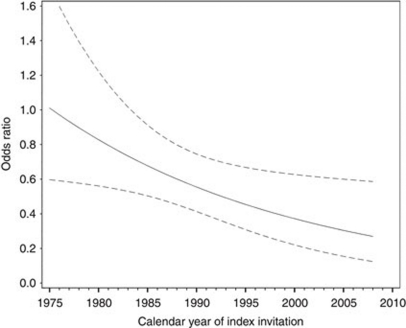
The OR of breast cancer death for screened *vs* unscreened women invited in the period 1975–2008. The line represents the OR along the continuum of calendar year of screening invitation; the dotted lines represent the 95% confidence interval.

Detailed analysis of the influence of calendar year of invitation showed a trend of increasing effectiveness of breast cancer screening over time (1975–2008) (Figure 1); *P*-value for interaction=0.02.

## Discussion

The results of our study show an increase in impact of mammographic service screening on the prevention of breast cancer death over time. There are a number of possible explanations for this increase in effectiveness. There have been significant improvements in mammographic screening and treatment over the last 30 years. However, methodological issues (confounding- and self-selection bias) may have influenced our results. We will discuss each of these points consecutively.

First, we believe that improvements in the quality of service screening (Hendrick *et al*, 2002; Yaffe *et al*, 2008; Ichikawa *et al*, 2010), that is, progressions in quality assurance, training of radiographers and radiologists and advances in mammography techniques, have had an effect on the growing benefit of screening. The introduction of an anti-scatter grid for mammography, a radiation exposure dispenser, the daylight system and improvements towards smaller focal spots have led to higher image quality with less radiation exposure (Yaffe *et al*, 2008).

Second, multidisciplinary teams have been working on the assessment of recalled women and treatment of patients since the start of the screening programme (Holland *et al*, 2007). Improvements in breast cancer treatment during the course of our study period have also resulted in a greater combined benefit of early detection and treatment. Since the 1970s, the use of chemotherapy and hormonal therapy after surgery has increased. In the Netherlands, this occurred predominantly between 1975 and 1990 (Vervoort *et al*, 2004). A meta-analysis has shown that adjuvant treatment of early stage breast cancer reduces breast cancer mortality (Early Breast Cancer Trialists' Collaborative Group, 2005). This overview indicates that chemotherapy at an early stage of the disease reduced breast cancer mortality by 20% in women aged 50–69. Furthermore, in patients with oestrogen receptor positive breast cancer, chemotherapy followed by tamoxifen or the use of tamoxifen alone caused an even greater breast cancer mortality reduction: about 31%. The success of adjuvant treatment for early stage breast cancer emphasises the importance of the synergy between early detection and early treatment (Berry *et al*, 2005).

Third, confounding bias could have had a role in our results, but we believe its influence on our effect estimates is marginal. The anticipated strong relation between a woman's age and the occurrence of breast cancer death, and the age-related participation in our screening programme, prompted us to correct for age at invitation.

We considered to what extent residual confounding bias remains after having addressed the influence of age. One candidate may be mammographic density, which in itself is an important risk factor for breast cancer (Boyd *et al*, 2007). However, the strong specific mammographic appearance composed of >75% of glandular tissue and stroma is only prevalent in about 5% of the post-menopausal women (Pisano *et al*, 2005). A correction for age also implies an indirect correction for mammographic density, because of the high correlation between mammographic density and age (Groenwold *et al*, 2010).

Other risk factors for breast cancer like obesity, socioeconomic status, nulliparity, late age at menopause, early age at menarche and family history show a 1.5–4-fold relative risk of breast cancer at most (Amir *et al*, 2010). Using sensitivity analysis (Schlesselman, 1978) we developed realistic scenarios of prevalence and strength of these risk factors on screened and not screened groups, and explored the impact of residual confounding bias. The results confirmed that a correction for residual confounding beyond age caused by these factors does not produce a major shift in our estimated OR. For instance, if a risk factor or risk profile with a relative risk of four is present in 10% of the screened women compared with 20% in the unscreened women, then our apparent OR of 0.35 would be adjusted to 0.43. Our effect estimate will only weaken in an extreme situation where a combination of strong risk factors is much less present among screened women compared with unscreened women.

Finally, related to the issue of confounding, is bias because of self-selection. Mammographic screening may seem more effective than it in fact is if women who participate in screening programmes have a lower background risk of dying from breast cancer. In the literature, contradictory results have been noted with regard to the direction and magnitude of self-selection bias. Where Friedman and Dubin (1991) found that screened women were at higher baseline risk for breast cancer death, Moss (1991) found the opposite.

To obtain a fair estimate of the amount of self-selection, the ratio of the breast cancer death among not-invited women and non-participants has to be calculated (Duffy *et al*, 2002). In our study, we were not able to calculate an estimate for self-selection, as we did not have an uninvited group for the main part of our study period. Nevertheless, we have two reasons for believing that the influence of self-selection bias in our results was only minor. First, during the early years, Verbeek *et al* (1984) performed a geographical comparison on breast cancer incidence rates and found no evidence of self-selection bias. Second, recently we (Paap *et al*, 2010a) quantified the extent of self-selection bias for a region close to Nijmegen. The resulting correction factor of 0.84 (95% CI=0.58–1.21) indicates a lower background risk in women who do not attend screening. When we applied this factor to the formula described by Duffy *et al* (2002), our OR of 0.35 changed to 0.28. Since both studies showed no major influence of self-selection bias and because we had a constant participation rate in our programme, we expect no change in the amount of this bias over time.

In the literature many different estimates on the preventive effect of breast cancer screening have been published. It is important to consider that study design and method of analysis contribute greatly to these differences. More than two decades ago, trials were performed in a ‘laboratory’ setting, whereas cohort and case–referent designs are used to evaluate real-life current screening practice. In trials, non-compliance in the invitation arm, and contamination, that is, screening examination in the control (not invited) arm cause an underestimation of the actual screening effect (Demissie *et al*, 1998). In cohort studies, differences in trends of breast cancer mortality are compared for screened and unscreened groups. A recent study on the Norwegian screening programme reported, after an average follow-up of 2.2 years, a seemingly disappointing 10% breast cancer mortality reduction because of screening (Kalager *et al*, 2010). On the basis of the diverging trends in mortality over time, as was demonstrated in a study from Sweden showing a 14% mortality reduction after 10 years in the age group 40–49 (Jonsson *et al*, 2000), and a 29% reduction after 16 years (Hellquist *et al*, 2011), the Norwegian results can actually be regarded as very promising.

In comparison with cohort studies, the case–referent design does not allow for estimating relative or absolute risks in breast cancer mortality. The advantage of the case–referent approach is that it directly links a woman's cause of death with her screening history. Therefore, we can accurately estimate the OR of screened *vs* unscreened women in the relevant time frame of screening invitation during the detectable preclinical period. As such, the OR is the mortality in screened *vs* not screened women. Case–referent studies from the England, Italy and Iceland, where screening started in the 1990s, showed a mortality reduction ranging from 41 to 65% (Gabe *et al*, 2007; Allgood *et al*, 2008; Puliti *et al*, 2008). In general, the design used in these studies is similar to ours (Paap *et al*, 2010b). The strength of our study is that we investigated temporal trends in screening effectiveness over time between 1975 and 2008.

In conclusion, we report on a strong and steady increase in the effectiveness of service screening on breast cancer mortality across the period 1975–2008, resulting in a 65% breast cancer mortality reduction in 1992–2008 compared with a 28% reduction in 1975–1991. Our findings demonstrate that mammographic screening has become more effective over time.

## Figures and Tables

**Table 1:** The effectiveness of mammographic screening on breast cancer mortality expressed by odds ratios, according to calendar period of index-invitation at screening and corrected for age at invitation

**Calendar period of index-invitation**	**Cases screened (unscreened)**	**Referents screened (unscreened)**	**Odds ratio (95% CI)**
1975–2008	191 (91)	1089 (321)	0.65 (0.49–0.87)
1975–1991	90 (40)	501 (149)	0.72 (0.47–1.09)
1992–2008	29 (23)	202 (58)	0.35 (0.19–0.64)

Abbreviation: CI=confidence interval.

## References

[bib1] Allgood PC, Warwick J, Warren RM, Day NE, Duffy SW (2008) A case-control study of the impact of the East Anglian breast screening programme on breast cancer mortality. Br J Cancer 98: 206–209 18059396 10.1038/sj.bjc.6604123 PMC2359716

[bib2] Amir E, Freedman OC, Seruga B, Evans DG (2010) Assessing women at high risk of breast cancer: a review of risk assessment models. J Natl Cancer Inst 102: 680–691 20427433 10.1093/jnci/djq088

[bib3] Autier P, Boniol M, LaVecchia C, Vatten L, Gavin A, Hery C, Heanue M (2010) Disparities in breast cancer mortality trends between 30 European countries: retrospective trend analysis of WHO mortality database. BMJ 341: c3620 20702548 10.1136/bmj.c3620 PMC2920378

[bib4] Becker N, Altenburg HP, Stegmaier C, Ziegler H (2007) Report on trends of incidence (1970–2002) of and mortality (1952–2002) from cancer in Germany. J Cancer Res Clin Oncol 133: 23–35 16896882 10.1007/s00432-006-0142-4 PMC12160771

[bib5] Berry DA, Cronin KA, Plevritis SK, Fryback DG, Clarke L, Zelen M, Mandelblatt JS, Yakovlev AY, Habbema JD, Feuer EJ (2005) Effect of screening and adjuvant therapy on mortality from breast cancer. N Engl J Med 353: 1784–1792 16251534 10.1056/NEJMoa050518

[bib6] Boyd NF, Guo H, Martin LJ, Sun L, Stone J, Fishell E, Jong RA, Hislop G, Chiarelli A, Minkin S, Yaffe MJ (2007) Mammographic density and the risk and detection of breast cancer. N Engl J Med 356: 227–236 17229950 10.1056/NEJMoa062790

[bib7] Broeders MJM, Verbeek ALM (2005) Mammographic screening only matters in the detectable preclinical period of breast cancer. J Med Screen 12: 107 15949123 10.1258/0969141053908267

[bib8] Demissie K, Mills OF, Rhoads GG (1998) Empirical comparison of the results of randomized controlled trials and case-control studies in evaluating the effectiveness of screening mammography. J Clin Epidemiol 51: 81–91 9474068 10.1016/s0895-4356(97)00243-6

[bib9] Duffy SW, Cuzick J, Tabár L, Vitak B, Chen THH, Yen MF, Smith RA (2002) Correcting for non-compliance bias in case-control studies to evaluate cancer screening programmes. J R Statist Soc Ser C Appl Stat 51(Part 2): 235–243

[bib10] Early Breast Cancer Trialists' Collaborative Group (EBCTCG) (2005) Effects of chemotherapy and hormonal therapy for early breast cancer on recurrence and 15-year survival: an overview of the randomised trials. Lancet 365: 1687–1717 15894097 10.1016/S0140-6736(05)66544-0

[bib11] Friedman DR, Dubin N (1991) Case-control evaluation of breast cancer screening efficacy. Am J Epidemiol 133: 974–984 2035508 10.1093/oxfordjournals.aje.a115817

[bib12] Gabe R, Duffy SW (2005) Evaluation of service screening mammography in practice: the impact on breast cancer mortality. Ann Oncol 16(Suppl 2): 153–162 10.1093/annonc/mdi718 15958448

[bib13] Gabe R, Tryggvadottir L, Sigfusson BF, Olafsdottir GH, Sigurdsson K, Duffy SW (2007) A case-control study to estimate the impact of the Icelandic population-based mammography screening program on breast cancer death. Acta Radiol 48: 948–955 18080359 10.1080/02841850701501725

[bib14] Greenland S, Thomas DC (1982) On the need for the rare disease assumption in case-control studies. Am J Epidemiol 116: 547–553 7124721 10.1093/oxfordjournals.aje.a113439

[bib15] Groenwold RH, Nelson DB, Nichol KL, Hoes AW, Hak E (2010) Sensitivity analyses to estimate the potential impact of unmeasured confounding in causal research. Int J Epidemiol 39: 107–117 19948779 10.1093/ije/dyp332

[bib16] Hellquist BN, Duffy SW, Abdsaleh S, Bjorneld L, Bordas P, Tabar L, Vitak B, Zackrisson S, Nystrom L, Jonsson H (2011) Effectiveness of population-based service screening with mammography for women ages 40 to 49 years: evaluation of the Swedish Mammography Screening in Young Women (SCRY) cohort. Cancer 117: 714–722 20882563 10.1002/cncr.25650

[bib17] Hendrick RE, Klabunde C, Grivegnee A, Pou G, Ballard-Barbash R (2002) Technical quality control practices in mammography screening programs in 22 countries. Int J Qual Health Care 14: 219–226 12108532 10.1093/oxfordjournals.intqhc.a002613

[bib18] Héry C, Ferlay J, Boniol M, Autier P (2008) Quantification of changes in breast cancer incidence and mortality since 1990 in 35 countries with Caucasian-majority populations. Ann Oncol 19: 1187–1194 18325921 10.1093/annonc/mdn025

[bib19] Holland R, Rijken H, Hendriks JHCL (2007) The Dutch population-based mammography screening: 30-year experience. Breast Care 2: 12–18

[bib20] Ichikawa LE, Barlow WE, Anderson ML, Taplin SH, Geller BM, Brenner RJ (2010) Time trends in radiologists' interpretive performance at screening mammography from the community-based Breast Cancer Surveillance Consortium, 1996–2004. Radiology 256: 74–82 20505059 10.1148/radiol.10091881 PMC2897687

[bib21] Jonsson H, Tornberg S, Nystrom L, Lenner P (2000) Service screening with mammography in Sweden – evaluation of effects of screening on breast cancer mortality in age group 40–49 years. Acta Oncol 39: 617–623 11093370 10.1080/028418600750013302

[bib22] Jorgensen KJ, Zahl PH, Gotzsche PC (2010) Breast cancer mortality in organised mammography screening in Denmark: comparative study. BMJ 340: c1241 20332505 10.1136/bmj.c1241 PMC2844939

[bib23] Kalager M, Zelen M, Langmark F, Adami HO (2010) Effect of screening mammography on breast-cancer mortality in Norway. N Engl J Med 363: 1203–1210 20860502 10.1056/NEJMoa1000727

[bib24] Levi F, Bosetti C, Lucchini F, Negri E, La VC (2005) Monitoring the decrease in breast cancer mortality in Europe. Eur J Cancer Prev 14: 497–502 16284493 10.1097/00008469-200512000-00002

[bib25] Miettinen O (1976) Estimability and estimation in case-referent studies. Am J Epidemiol 103: 226–235 1251836 10.1093/oxfordjournals.aje.a112220

[bib26] Moss SM (1991) Case-control studies of screening. Int J Epidemiol 20: 1–6 2066205 10.1093/ije/20.1.1

[bib27] Nelson HD, Tyne K, Naik A, Bougatsos C, Chan BK, Humphrey L (2009) Screening for breast cancer: an update for the U.S. Preventive Services Task Force. Ann Intern Med 151: 727–742 19920273 10.1059/0003-4819-151-10-200911170-00009 PMC2972726

[bib28] Otten JDM, Broeders MJM, Fracheboud J, Otto SJ, de Koning HJ, Verbeek ALM (2008) Impressive time-related influence of the Dutch screening programme on breast cancer incidence and mortality, 1975–2006. Int J Cancer 123: 1929–1934 18688863 10.1002/ijc.23736

[bib29] Otten JDM, Van Dijck JAAM, Peer PG, Straatman H, Verbeek ALM, Mravunac M, Hendriks JH, Holland R (1996) Long term breast cancer screening in Nijmegen, the Netherlands: the nine rounds from 1975–92. J Epidemiol Community Health 50: 353–358 8935470 10.1136/jech.50.3.353 PMC1060295

[bib30] Paap E, Holland R, den Heeten GJ, van Schoor G, Botterweck AA, Verbeek ALM, Broeders MJM (2010a) A remarkable reduction of breast cancer deaths in screened versus unscreened women: a case-referent study. Cancer Causes Control 21: 1569–1573 20512656 10.1007/s10552-010-9585-7

[bib31] Paap E, Verbeek ALM, Puliti D, Paci E, Broeders MJM (2010b) Breast cancer screening case-control study design: impact on breast cancer mortality. Ann Oncol; e-pub ahead of print 5 October 2010, doi:10.1093/annonc/mdq447 10.1093/annonc/mdq447 20924073

[bib32] Pisano ED, Gatsonis C, Hendrick E, Yaffe M, Baum JK, Acharyya S, Conant EF, Fajardo LL, Bassett L, D'Orsi C, Jong R, Rebner M (2005) Diagnostic performance of digital versus film mammography for breast-cancer screening. N Engl J Med 353: 1773–1783 16169887 10.1056/NEJMoa052911

[bib33] Puliti D, Miccinesi G, Collina N, De Lisi V, Federico M, Ferretti S, Finarelli AC, Foca F, Mangone L, Naldoni C, Petrella M, Ponti A, Segnan N, Sigona A, Zarcone M, Zorzi M, Zappa M, Paci E (2008) Effectiveness of service screening: a case-control study to assess breast cancer mortality reduction. Br J Cancer 99: 423–427 18665188 10.1038/sj.bjc.6604532 PMC2527797

[bib34] Rothman KJ, Greenland S, Lash TL (2008) Modern Epidemiology. Wolters Kluwer Health/Lippincott Williams & Wilkins: Philadelphia

[bib35] Schlesselman JJ (1978) Assessing effects of confounding variables. Am J Epidemiol 108: 3–8 685974

[bib36] Shapiro S, Coleman EA, Broeders MJM, Codd MB, de Koning HJ, Fracheboud J, Moss S, Paci E, Stachenko S, Ballard Barbash R (1998) Breast cancer screening programmes in 22 countries: current policies, administration and guidelines. Int J Epidemiol 27: 735–742 9839727 10.1093/ije/27.5.735

[bib37] Verbeek ALM, Broeders MJM (2010) Evaluation of cancer service screening: case referent studies recommended. Stat Methods Med Res 19: 487–505 20356858 10.1177/0962280209359856

[bib38] Verbeek ALM, Hendriks JHCL, Holland R, Mravunac M, Sturmans F, Day NE (1984) Reduction of breast cancer mortality through mass screening with modern mammography. First results of the Nijmegen project, 1975–1981. Lancet 1: 1222–1224 6144933 10.1016/s0140-6736(84)91703-3

[bib39] Vervoort MM, Draisma G, Fracheboud J, van de Poll-Franse LV, de Koning HJ (2004) Trends in the usage of adjuvant systemic therapy for breast cancer in the Netherlands and its effect on mortality. Br J Cancer 91: 242–247 15213715 10.1038/sj.bjc.6601969 PMC2409826

[bib40] Weiss NS, McKnight B, Stevens NG (1992) Approaches to the analysis of case-control studies of the efficacy of screening for cancer. Am J Epidemiol 135: 817–823 1595681 10.1093/oxfordjournals.aje.a116368

[bib41] Yaffe MJ, Mainprize JG, Jong RA (2008) Technical developments in mammography. Health Phys 95: 599–611 18849694 10.1097/01.HP.0000327648.42431.75

[bib42] Zahl PH, Maehlen J (2005) Reduction in mortality from breast cancer: decrease with screening was marked in younger age group. BMJ 330: 1024 10.1136/bmj.330.7498.1024 PMC557158 15860834

